# Interactions between glycopyrronium and indacaterol on cholinergic neurotransmission and contractile response in bovine trachealis

**DOI:** 10.1186/s12931-017-0627-5

**Published:** 2017-07-28

**Authors:** Michele Baroffio, Giovanni Barisione, Vito Brusasco

**Affiliations:** 1 Dipartimento di Medicina Interna e Specialità Mediche, Università di Genova, Viale Benedetto XV 6, 16132 Genoa, Italy; 2 Ospedale Policlinico San Martino, Largo R. Benzi 10, 16132 Genoa, Italy

**Keywords:** Airway smooth muscle, Acetylcholine, Long-acting muscarinic antagonist, Long-acting β-adrenoceptor agonist

## Abstract

**Background:**

Muscarinic-receptor antagonists and β-adrenoceptor agonists are used, alone or in combination, as first-line treatment for chronic obstructive pulmonary disease. Both drugs decrease airway smooth muscle tone by post-junctional mechanisms but they may have opposing effects on pre-junctional acetylcholine (ACh)-release.

**Methods:**

We studied the effects of the muscarinic-receptor antagonist glycopyrronium (GLY), the β-adrenoceptor agonist indacaterol (IND) and their combination on electrically-induced ACh-release and contractile response in isolated bovine trachealis. Data were analyzed by paired t-test and analysis of variance for repeated or independent measures with Newmann-Keuls post-hoc test when appropriate.

**Results:**

GLY 10^−8^ M decreased contractile response by 19 ± 6% (*p* = 0.010) without altering ACh-release. GLY 10^−7^ M and 10^−6^ M almost abolished contractile responses even if the ACh-release was increased by 27 ± 19% (*p* < 0.001) and 20 ± 8% (*p* = 0.004), respectively. IND 10^−7^ M had no significant effects on contractile response and ACh-release, whereas IND 10^−6^ M reduced contractile response by 24 ± 12% (*p* = 0.002) without altering ACh-release. IND 10^−5^ M decreased contractile response by 51 ± 17% (*p* < 0.001) and ACh-release by 22 ± 11% (*p* = 0.004). Co-incubation with GLY 10^−8^ M and IND 10^−7^ M did not alter ACh-release but inhibited contractile response by 41 ± 8% (*p* < 0.001). The latter effect was greater than with GLY 10^−8^ M, or IND 10^−7^ M, or IND 10^−6^ M given separately (*p* < 0.001 for all). The increment of ACh-release caused by GLY was attenuated by IND 10^−5^ M, though this did not affect contractile response.

**Conclusions:**

At equimolar concentration, GLY alone attenuates airway smooth muscle contraction more than IND, despite an increased ACh-release. Combination of GLY with IND at submaximal concentrations has more than additive effect suggesting a synergistic post-junctional effect. Adding GLY to IND provides a greater inhibitory effect on airway smooth muscle contraction than increasing IND concentration.

**Electronic supplementary material:**

The online version of this article (doi:10.1186/s12931-017-0627-5) contains supplementary material, which is available to authorized users.

## Background

In airway smooth muscle, the acetylcholine (ACh)-release and the contractile response are thought to be modulated both by cholinergic muscarinic (M) receptors [1–7] and β_2_-adrenoceptors [8, 9]. Physiologically, the airway smooth muscle contractile response is enhanced by ACh acting on post-junctional M_3_ receptors and inhibited by catecholamine acting on post-junctional β_2_-adrenoceptors. Therefore, M-receptor antagonists and β_2_-adrenoceptor agonists are used alone or in combination for treatment of chronic obstructive pulmonary disease (COPD). However, their effects at pre-junctional level are opposing, with muscarinic-receptor antagonists increasing [1–7] and β_2_-adrenoceptor agonists decreasing ACh-release [8, 9] from post-ganglionic nerves.

The relaxing effect of M-antagonists on airway smooth muscle might be partially offset by stimulation of pre-junctional M_2_ or M_4_ auto-receptors [3, 5, 6] resulting in an increase of ACh-release. By contrast, β_2_-adrenoceptor agonists significantly inhibit ACh-release, thus enhancing their post-junctional relaxing effects [8]. Therefore, one can speculate that the inhibitory pre-junctional effect of β_2_-adrenoceptor agonists on ACh-release may counteract the stimulatory pre-junctional effect of M-receptor antagonists by functional antagonism.

On the other hand, the relaxing effect of β_2_-adrenoceptor agonists may be partially offset by stimulation of post-junctional M_2_-receptors, via the receptor-coupled G_i_-protein inhibiting adenylyl-cyclase [10]. Therefore, non-selective M-receptor antagonists may enhance the effect of β_2_-adrenoceptor agonists by a synergistic effect at post-junctional level, as suggested by data in airway [11] and ocular [12] smooth muscle. Were this the case, then it may represent a rationale for clinical use of combined β_2_-adrenoceptor agonists and muscarinic antagonists at doses lower than those of the individual components used alone.

The aims of this study in vitro were to *1)* compare the inhibitory effects of two long-acting bronchodilators, the M-antagonist glycopyrronium (GLY) and the β_2_-adrenoceptor agonist indacaterol (IND) on airway smooth muscle contraction in isolated bovine trachealis, *2)* evaluate the pre-junctional effects of GLY and IND on ACh-release, and 3) investigate whether GLY and IND have additive or synergistic effects on ACh-release and contractile response.

## Methods

### Tissue preparation

Fifty bovine tracheas were obtained from two local abattoirs. The ethical approval was not required as we were not involved in the care or killing of the animals. After death the tracheas were removed and immersed in chilled (4 °C) physiologic salt solution (PSS) of the following composition (MgSO_4_ 0.8 mM, KH_2_PO_4_ 1.2 mM, KCl 3.4 mM, CaCl_2_ 2.4 mM, NaCl 110.5 mM, NaHCO_3_ 25.7 mM, and dextrose 5.6 mM). The mucosa, including epithelium, was removed. Twenty-two tracheas were used on the same day of animals’ death, 20 after 24-h and 8 after 48-h storage in aerated (95% O_2_, 5% CO_2_) and chilled (4 °C) PSS.

### General procedures

#### Characterization of GLY antagonism in bovine trachealis

Tracheal strips were mounted in 25-ml glass-jacketed tissue baths, containing aerated (95% O_2_ and 5% CO_2_) and warm (37 °C) PSS. Strips were treated with propranolol 10^−6^ M and hexamethonium 10^−5^ M to prevent β-adrenergic and nicotinic receptor activation. The strips were suspended between 2 rectangular (1 × 4 cm) platinum electrodes. The lower muscle end was tied to a stationary hook and the upper to a calibrated force transducer (MODEL FT03, Grass Medical Instruments, Fullerton, CA) mounted on a micromanipulator. Forces were continuously recorded using a multichannel thermal array recorder (Gould model TA, 4000, Valley View, OH) and an Arduino Due microcontroller board USB-connected to a laptop. Strips were equilibrated for 2 h while electrical field stimulation (15 V, 25 Hz, 0.5 ms) was delivered by a direct current amplifier (Mayo Clinic, Section of Engineering, Rochester, MN) triggered by a stimulator (S44, Grass Medical Instruments, Quincy, MA) every 5 min for 30 s. The muscles were stretched between stimulations to the length at which they produced their maximal and consistent force. This was the reference length (L_ref_) [13] which was not altered during each experiment. In 48 muscle strips from 6 animals, frequency response curves (from 0.06 to 64 Hz, every 5 min, in random order) to electrical field stimulation (15 V, 0.5 ms, 30 s) where first obtained. After this control assessment, muscles where first washed, then one muscle from each animal was used as control, while other 7 strips were incubated with a single dose of GLY (10^−9^-10^−6^ M, half-Log increments) for 45 min. A second frequency response curve was then obtained. In 56 muscle strips from 7 animals, dose–response curves to exogenous ACh (10^−9^-10^−4^ M, cumulative half-Log increments) was first obtained. After this control assessment, muscles were washed until their resting tension was achieved. Then one strip from each animal was used as control, while other 7 strips were incubated with a single dose of GLY (10^−9^-10^−6^ M, half-Log increments) for 45 min. A second ACh dose–response curve was then obtained (10^−9^-10^−1^ M, cumulative half-Log increments). At the end of each study, muscles were blotted dry and weighed.

#### [^3^H]-ACh-release and contractile response induced by electrical stimulation

Two trachealis strips from each animal were suspended in 2-mL water-jacketed tissue baths containing aerated (95% O_2_ and 5% CO_2_) and warm (37 °C) PSS. The muscle ends were tied to two different platinum wire electrodes connected to a micromanipulator and a force transducer (LC 4001, G0120, Litra Co, Japan), respectively. Both muscles were superfused (1 mL/min) with PSS using a calibrated roller pump (Gilson® Miniplus 3, Villiers Le Bel, France). Choline 10^−6^ M and indomethacin 10^−5^ M were added to reduce [^3^H]-choline deposition on bath walls and plastic tubes and block prostaglandin synthesis, respectively. Isometric forces were continuously and simultaneously recorded (Linseis L 250 E recorder, Selby, Germany). The strips were electrically-contracted (25 V, 25 Hz, 0.5 ms) every 5 min for 30 s. The electrical stimuli were provided by a stimulator (S44, Grass Medical Instruments, Quincy, MA). Between stimulations, strips were stretched to L_ref_ [13]. L_ref_ was not altered during the studies. The muscles were then superfused (2 mL/min) with aerated warm PSS containing 1.4 μCi/mL [^3^H]-choline (specific activity 78.3 Ci/mM). During equilibration with [^3^H]-choline both muscles were continuously electrically-stimulated (25 V, 25 Hz, 0.5 ms) for 30 min to enhance neuronal uptake of [^3^H]-choline. Then muscles were washed (20 mL/min) for 90-min with aerated PSS added with hemicholinum-3 10^−5^ M to block neuronal re-uptake of [^3^H]-choline. The PSS flow was then reduced to l mL/min and collections of superfusates were begun. This time is referred to as time zero (t_0_); t_n_ indicates minutes after t_0_. Superfusates were collected separately but simultaneously from both muscles for 3 min periods in vials containing 14 mL of liquid scintillation cocktail (Ultima Gold™, Perkin Elmer, Waltham, MA). Collections were interrupted twice for 10 min allowing the incubation with test compounds. At the end of the studies muscles were blotted dry and weighed. Each vial was assayed for radioactivity by liquid scintillation counting (LS 6500 multipurpose scintillation counter, Beckman Instruments, Fullerton CA) three times for 5 min and the average used for statistical analysis. Scintillation counts per minute were divided for the counting efficiency calculated from a quench curve, and disintegrations per minute were obtained. Disintegrations per stimulus were then determined from the areas bound by spontaneous and electrically induced [^3^H]-ACh-release.

### Experimental protocol

#### Effects of GLY, IND, or both on electrically-induced contractile response and [^3^H]-ACh-release (Fig. 1)

One muscle from each animal was used as control and received no study drugs. For studies with GLY alone, strips were pre-treated with propranolol 10^−6^ M to prevent β-adrenergic activation. After control measurements of electrically-induced (25 V, 4 Hz, 0.5 ms, 3 min) contractile response and [^3^H]-ACh-release at t_18_, test muscles were incubated at t_39_ with study drugs (GLY 10^−9^ M, *n* = 7 or 10^−7^ M, *n* = 6), or (IND 10^−8^ M, *n* = 6 or 10^−6^ M, *n* = 6) or (GLY 10^−9^ M + IND 10^−8^ M, *n* = 6) or (GLY 10^−7^ M + IND 10^−6^ M, *n* = 6) and a second electrical stimulation applied at t_67_. Test muscles were then incubated at t_88_ with study drugs (GLY 10^−8^ M, *n* = 7 or 10^−6^ M, *n* = 6) or (IND 10^−7^ M, *n* = 6 or 10^−5^ M, *n* = 6) or (GLY 10^−8^ M + IND 10^−7^ M, *n* = 6), or (GLY 10^−6^ M + IND 10^−5^ M, *n* = 6) and a third electrical stimulation applied at t_116_. At t_137_ superfusate collections were stopped.

**Fig. 1:**
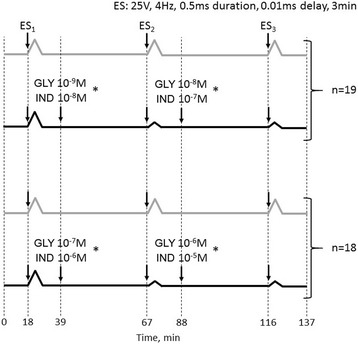
Temporal sequences of studies performed to evaluate the effects of glycopyrronium (GLY) and indacaterol (IND) alone or in combination (*) on electrically-evoked contractile response and [^3^H]-acetylcholine (ACh)-release. Grey and black lines are referred to control and test muscles, respectively

### Data analysis

**Fig. 2:**
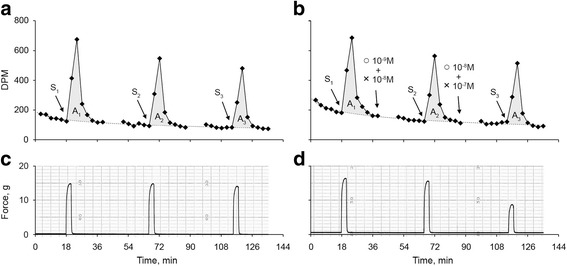
Example of [^3^H]-ACh-release (**a** and **b** panels) and isometric force (**c** and **d** panels) in response to electrical stimulation in control (**a** and **c** panels) and test (**b** and **d** panels) bovine trachealis. DPM are disintegrations per min. S_1_, S_2_, and S_3_ represent first, second, and third electrical stimulation, respectively. Dashed lines represent the extrapolated spontaneous [^3^H]-ACh-release. The grey areas (A_1_, A_2_, and A_3_) are the [^3^H]-ACh-release induced by the corresponding stimulations. The electrically-induced [^3^H]-ACh-release decrease after each stimulation (A_1_ > A_2_ > A_3_) suggesting pre-junctional neuronal depletion of [^3^H]-ACh. ♦ represent radioactivity measurements of superfusate by liquid scintillation counting. Combination of GLY(○) and IND (×) inhibited the electrically-induced contractile response without altering the [^3^H]-ACh-release

All contractile responses were determined as peak force minus basal tone measured at t_0_. The percent changes of [^3^H]-ACh-release and force at each n stimulation was calculated as [(A_n drug_/A_1 drug_)/(A_n control_/A_1 control_) − 1] • 100 and [(F_n drug_/F_1 drug_)/(F_n control_/F_1 control_) − 1] • 100 . A_1_ , A_n_ are the areas, expressed as disintegrations per stimulus, between [^3^H]-ACh-released upon electrical stimulation and spontaneous [^3^H]-ACh-release. F_1_, F_n_ are the corresponding forces (Fig. 2). The Bliss’ equation for drug independence [14, 15] was used to evaluate the difference between the expected effects based on GLY and IND when given separately and the observed effects when given in combination.

For statistical analysis, t-test for paired data and analysis of variance for repeated or independent measurements with Newmann-Keuls post-hoc test were used when appropriate. Data were analyzed and graphed using Statistica 6.0 (StatSoft, Inc., Tulsa, OK) and GraphPad Prism 6.01 (GraphPad Software, Inc., La Jolla, CA) software, respectively. *P* < 0.05 was considered to be statistically significant. Data are reported as means ± SD.

### Drugs

Indomethacin, dl-propranolol hydrochloride, hemicholinium-3 bromide, choline chloride, were purchased from Sigma Chemical (Milan, Italy). Methyl [^3^H]-choline was obtained from NEN™ Life Science Products, Inc. (Boston, MA), and scintillation cocktail Ultima Gold™ from Perkin Elmer (Waltham, MA). GLY and IND were provided by Novartis Pharma (Basel, CH). Indomethacin was dissolved in absolute ethanol and IND in dimethyl sulfoxide; all other compounds were dissolved in distilled water. Fresh solutions were prepared weekly and stored at 4 °C to be used within 7 days.

## Results

**Fig. 3:**
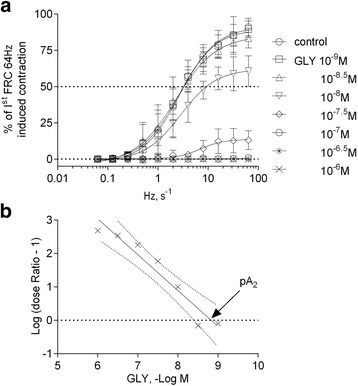
**a**: frequency-response curves (FRC) obtained in 48 bovine trachealis from 6 animal in the absence or presence of different GLY concentrations. Data (means ± SD) are expressed as percent of 64-Hz-induced contraction obtained for each trachealis strip during the first FRC. **b**: Schild regression to exogenous ACh obtained on 56 bovine trachealis from 7 animals. Regression line was calculated by linear regression analysis of mean values (x) obtained in 7 different experiments. Dashed lines define the 95% confidence limits and arrow is referred to the GLY pA_2_ value. The dose ratio is the concentration of ACh inducing half-maximal contraction (EC_50_) in the presence of a given concentration of GLY divided for the EC_50_ for ACh in the absence of GLY. GLY inhibited both exogenous ACh- and electrically-evoked contractions

One hundred and seventy-eight muscle strips from 50 animals were used, their physical and functional characteristics at baseline are reported in Additional file 1: Table S1. Neither GLY nor IND altered the muscle resting tone. GLY caused significant rightward shifts of response curves to both frequency and exogenous ACh at concentration larger or equal to 10^−8^ M (*p* < 0.001, Fig. 3). Schild regression analysis yielded a pA_2_ of 8.68 ± 0.21 and a slope of −1.06 ± 0.07 which was not significantly different from unity (*p* = 0.267) suggesting competitive antagonism [16] (Fig. 3). The [^3^H]-ACh-release consistently decreased in all muscle with time (Fig. 2) due to progressive depletion of radiolabeled ACh and inhibition of neural choline uptake induced by hemicholinium-3 [3, 5–8, 17–19].

### Effects of GLY on electrically-evoked contractile response and [^3^H]-ACh-release (Fig. 4)

GLY had no significant (*p* > 0.726) effect on either contractile response or [^3^H]-ACh-release at 10^−9^ M (−2 ± 3% and −3 ± 5%, respectively). At 10^−8^ M, GLY significantly reduced the contractile response (−19 ± 6%, *p* = 0.010) without significantly altering [^3^H]-ACh-release (6 ± 11%, *p* = 0.604). At 10^−7^ M and 10^−6^ M the contractile responses were significantly reduced (−97 ± 3%, *p* < 0.001 and −99 ± 1%, *p* < 0.001, respectively). These reductions were associated with significant increments of [^3^H]-ACh-release at 10^−7^ M and 10^−6^ M (27 ± 19%, *p* < 0.001 and 20 ± 8%, *p* = 0.004, respectively).

**Fig. 4:**
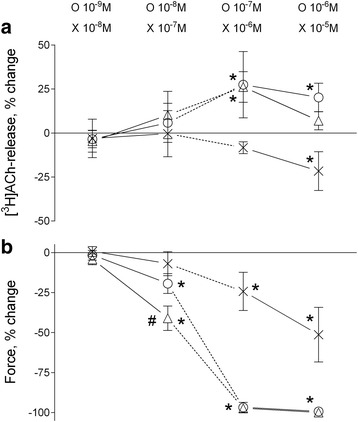
Effect of GLY (○, *n* = 13) and IND (×, *n* = 12) alone or combined (Δ, n=12) on electrically-induced [^3^H]-ACh-release and force (**a** and **b** panels, respectively). Continuous and interrupted lines indicate paired and unpaired data, respectively. Data are means ± SD; *, significantly different from zero (*p* < 0.05); #, significantly different from GLY 10^−8^ M (*p* < 0.001), IND 10^−7^ M, and IND 10^−6^ M (*p* < 0.001) alone

### Effects of IND on electrically-evoked contractile response and [^3^H]-ACh-release (Fig. 4)

IND had no significant (*p* > 0.607) effects on either contractile response or [^3^H]-ACh-release at 10^−8^ M (1 ± 3% and −2 ± 6%, respectively) and 10^−7^ M (−7 ± 7% and 0 ± 13%, respectively). At 10^−6^ M, IND significantly reduced the contractile response (−24 ± 12%, *p* = 0.002) without significantly altering [^3^H]-ACh-release (−8 ± 3%, *p* = 0.396). At 10^−5^ M the contractile response was reduced (−51 ± 17%, *p* < 0.001) and this was associated with a decrease of [^3^H]-ACh-release (−22 ± 11%, *p* = 0.004).

### Effects of combined GLY and IND on electrically-evoked contractile response and [^3^H]-ACh-release (Fig. 4)

Co-incubation with GLY 10^−9^ M and IND 10^−8^ M had no significant (*p* > 0.970) effects on either contractile response or [^3^H]-ACh-release (−4 ± 3% and −5 ± 6%, respectively). Co-incubation with GLY 10^−8^ M and IND 10^−7^ M inhibited the contractile response (−41 ± 8%, *p* < 0.001) significantly more than GLY 10^−8^ M alone (−19 ± 6%, *p* < 0.001), or IND 10^−7^ M alone (−7 ± 7%, *p* < 0.001), or IND 10^−6^ M alone (−24 ± 12%, *p* < 0.001) without significantly altering [^3^H]-ACh-release (10 ± 13%, *p* = 0.368). With GLY 10^−7^ M and IND 10^−6^ M neither contractile response nor [^3^H]-ACh-release significantly differed from those with GLY 10^−7^ M alone (−97 ± 3% vs *−* 97 ± 3%, *p* = 0.899 and 26 ± 9% vs 27 ± 19%, *p* = 0.775, respectively). With GLY 10^−6^ M and IND 10^−5^ M the contractile responses were not significantly different from that with GLY 10^−6^ M alone (−100 ± 0.1% vs −99 ± 1% *p* = 0.893) while the increase of [^3^H]-ACh-release was significantly reduced (7 ± 5% vs 20 ± 8%, *p* = 0.019).

### Comparison between expected and observed effects of GLY and IND combination

For [^3^H]-ACh-release none of the observed effects of combination was significantly different from those expected based on their effects when given separately (*p* > 0.622), suggesting additive effects. For contractile response, the observed effect with the combination of GLY 10^−8^ M and IND 10^−7^ M was significantly greater than the expected (41 ± 8% vs. 28 ± 10% *p* = 0.002), suggesting a more than additive effect.

## Discussion

The main findings of the present study are that *1)* on equimolar basis GLY had a significantly larger inhibitory effect on electrically﻿-﻿ induced contraction of airway smooth muscle than IND, even if it caused an increase in ACh-release, *2)* the combination of GLY 10^−8^ M with IND 10^−7^ M had a more than additive effect in inhibiting the contractile response, without significantly altering [^3^H]-ACh-release, and *3)* adding GLY to IND had a greater effect than increasing the concentration of IND alone.

### Comments on methodology and study limitations

ACh-release was evaluated from the outflow of [^3^H]. The validity of this method for measuring [^3^H]-ACh has been demonstrated by Kilbinger et al. in 1991 [3]. The strength of this method is that it does not require cholinesterase inhibitors, thus avoiding unphysiological high concentrations of ACh resulting in M-receptor auto-inhibition. A potential problem of this technique is that the release of [^3^H]-ACh may not reflect accurately the release of endogenous ACh [18], but this has not been confirmed [19]. In order to measure quantitatively the electrically induced ACh-release, neuronal re-uptake of [^3^H]-choline was blocked by hemicholinium-3, resulting in [^3^H]-ACh depletion in postganglionic cholinergic nerve endings with each consecutive electric stimulation. The number of consecutive stimulations in each study was therefore limited to three. However, [^3^H]-ACh-release is affected by the magnitude of depletion with previous stimulations. If [^3^H]-ACh-release is altered by study drugs, depletion can either be increased or reduced with the second stimulation, thus leaving less or more [^3^H]-ACh available to be released with the third electrical stimulation. In the present study there were no significant differences in [^3^H]-ACh-release after second stimulation between control muscles and muscles treated with either GLY 10^−9^ M, IND 10^−8^ M, IND 10^−6^ M, or with GLY 10^−9^ M co-incubated with IND 10^−8^ M. With the highest GLY concentrations, the [^3^H]-ACh-release was greater than control, therefore there is a possibility that [^3^H]-ACh-release after GLY 10^−6^ M incubation might have been underestimated.

The frequency of 4 Hz for the assessment of electrically-induced [^3^H]-ACh-release and contractile response was chosen from frequency-response curves, where it caused a near half-maximal contraction. The concentrations of GLY and IND used in the electrically-induced [^3^H]-ACh-release and contractile response experiments were chosen from the frequency- and exogenous ACh-response curves characterizing GLY antagonism and from previous data with other β-adrenoceptor agonists [8], respectively.

Electrically-induced ACh-release may be attenuated in isolated bovine trachealis by stimulation of pre-junctional β_2_-adrenoceptor [8]. Thus, in experiments with GLY alone, propranolol was added. Activation of muscarinic receptors stimulates synthesis and release of prostaglandins, which in turn reduces ACh-release [4, 20, 21]. Thus, to avoid any confounding effect of prostaglandins, studies were done in the presence of indomethacin.

The present study was done using bovine trachealis. Therefore, any extrapolation of our data to human bronchi either in healthy subjects or in patients having COPD, with different β_2_-adrenoceptor and M-receptor density [22], should be draw cautiously. The choice of this tissue was based on the sufficient availability to conduct the large number of experiments necessary for the study and because muscarinic autoregulation [6] is present in this tissue similar to human airways [1, 2, 5].

Isometric contraction does not represent what happens in vivo, where airway smooth muscle contraction occurs under auxotonic conditions. However, this does not invalidate conclusions regarding interactions between drugs acting on different receptors.

### Comments on results

Long-acting β_2_-adrenoceptor agonists and cholinergic M-receptor antagonists are recommended by expert committees [23] and guidelines [24, 25] as the cornerstone treatment for COPD. No specific recommendations are given on the strategy to optimize the use of these drugs. One option is starting treatment with either one of them and, if not sufficient to control symptoms, increasing the dosage of the same drug or adding a second one; an alternative option is starting with low-dosage combination treatment. The M-receptor antagonist ipratropium caused a bronchodilator effect that was maximal in patients with bronchitis but not in those with bronchial asthma [26]. Based on this finding, it has long been thought that cholinergic tone and the amplifying effect of airway wall thickening are the main mechanisms for airway narrowing in COPD [26–29]. Because no difference was observed between ipratropium and a combination of fenoterol plus theophylline, the choice between β_2_-adrenoceptor agonists and M-receptor antagonists as monotherapy for COPD has been considered as a matter of side effects more than efficacy. If these data from isolated bovine airways can be extrapolated to human airways, then the results would suggest that muscarinic antagonists might represent the treatment of choice and the better strategy for monotherapy in COPD.

The superiority of GLY over IND in attenuating airway smooth muscle contraction might appear surprising owing to the expected opposing effects of these drugs on ACh-release from pre-junctional post-ganglionic nerves. As other available anti-muscarinic drugs, GLY is not selective for M_3_-receptor [30, 31], but it increases ACh-release by antagonizing also pre-junctional M_2_ and/or M_4_ receptors [3, 5, 6]. By contrast, β_2_-adrenoceptor agonists may reduce ACh-release by opening pre-junctional Ca^2+^-dependent K^+^ channels [8, 32]. In the present study, at concentration of GLY less than 10^−7^ M, electrically-induced ACh-release remained unaltered, whereas at concentrations of 10^−7^ M and 10^−6^ M the ACh-release was increased. This difference in response might be due to blocking ganglionic M_1_-receptors at low concentrations offsetting the effect on post-ganglionic M_2_-receptor at low but not high concentrations [33–35]. The increments of ACh-release with GLY or GLY + IND were on average between 20 and 27%. Based on a previous study using opioid agonists [7] and frequency-response curves (Additional file 1: Figure S1), we estimated that these changes would have resulted in changes of force between 8 and 10% in the absence of drugs acting at post-junctional level. These changes, however, cannot be quantitatively extrapolated to in vivo conditions for a series of reasons. First, the relationship between ACh-release and force is not linear and the frequency of vagal firing may be variably affected by different stimuli [36]. Second, changes in airway smooth muscle force translate into changes of linear length depending on opposing loads [27]. Finally, changes in linear length translate into changes in airway caliber depending on airway geometry [29]. It can be speculated that changes in force may translate into greater changes of airway caliber in COPD than healthy subjects because of the increased thickness of airway walls and the loss of lung elastic recoil [27–29].

Interestingly, the contractile responses were abolished by 10^−7^ M and 10^−6^ M concentrations of GLY, despite an increase of ACh-release. Presumably, complete blockade of post-junctional M_3_-receptor prevented a contractile response to the increased ACh-release. This is consistent with the absence of contractile non-cholinergic stimuli in bovine trachealis [6]. Conversely, at 10^−5^ M IND reduced ACh-release and, importantly, the net inhibitory effects on contractile responses were less than with GLY lower concentrations.

A major objective of the present study was to investigate whether the GLY-IND combination may have additive or more-than additive effects in reducing airway smooth muscle contraction and ACh-release. The combination of GLY 10^−8^ M with IND 10^−7^ M attenuated contractions more than GLY 10^−8^ M, or IND 10^−7^ M given separately, and the expected based on Bliss independent criterion [14, 15]. This more than additive effect in conjunction with the insignificant effects of IND 10^−7^ M alone on ACh-release suggest a synergistic effect at post-junctional level, rather than a functional antagonism at pre-junctional level.

M_2_-receptors are expressed not only pre-junctionally on nerves but also post-junctionally on airway smooth muscle cell membrane [37, 38]. Inhibition of post-junctional M_2_ receptors by muscarinic antagonist inhibits the receptor-coupled G_i_α subunit, thus favoring the adenylyl cyclase activity, the cAMP accumulation, and thereby the relaxant effect of β_2_-adrenoceptor agonists [39, 40]. Therefore, non-selective muscarinic antagonists may increase the relaxing effects of β_2_-adrenoceptor agonists. Indeed, in human bronchi a greater cAMP concentration has been observed with a GLY-IND combination than with IND alone [11].

An important finding of this study is that adding a low concentration (10^−8^ M) of GLY to IND 10^−7^ M had an inhibitory effect on airway smooth muscle contraction that was superior to increasing IND concentration by 10 times and not inferior to increasing it by 100 times.

## Conclusions

In isolated bovine trachealis, at equimolar concentration, the muscarinic antagonist GLY has a significant larger inhibitory effect on airway smooth muscle contraction than IND. Importantly, a combination of GLY with IND at submaximal concentrations can provide a significantly larger inhibition of contractile response than either drug alone without altering ACh-release, suggesting a synergistic post-junctional effect. Collectively, this study suggests that GLY may be the first-choice for monotherapy and low-dosage combination of GLY and IND may be preferable than increasing IND alone.

## Additional file

Additional file 1:
**Table S1.** Physical and baseline functional characteristics of bovine tracheal strips. **Figure S1.** Relationships between ACh-release and isometric force at ES frequencies of 1 (x), 2 (□), 4 (●), 8 (◊), and 16 (○) Hz in 4 separate experiments. Data are mean and SD of percent changes from 4 Hz; continuous thick line is the regression model of the form *y* = (*plateau* − *y*_0_ · [1 − *e* ^(−*k* · *x*)^]), interrupted lines are the 95% CI; *r* ^2^ = 0.887. (DOCX 175 kb)

## Abbreviations

A: Area
ACh: Acetylcholine
COPD: Chronic obstructive pulmonary disease
F: Force
GLY: Glycopyrronium
IND: Indacaterol
M: Muscarinic
PSS: Physiologic salt solution
