# Supplementary material for: Interactions between glycopyrronium and indacaterol on cholinergic neurotransmission and contractile response in bovine trachealis
Source: Respir Res. 2017 Jul 28;18:145. doi: 10.1186/s12931-017-0627-5 (PMC5534043; doi:10.1186/s12931-017-0627-5)
Supplement: Additional file 1: — Table S1. Physical and baseline functional characteristics of bovine tracheal strips. Figure S1. Relationships between ACh-release and isometric force at ES frequencies of 1 (x), 2 (□), 4 (●), 8 (◊), and 16 (○) Hz in 4 separate experiments. Data are mean and SD of percent changes from 4 Hz; continuous thick line is the regression model of the form y = (plateau − y 0 · [1 − e (−k · x)]), interrupted lines are the 95% CI; r 2 = 0.887. (DOCX 175 kb) [file 12931_2017_627_MOESM1_ESM.docx]

**Online supplement:**

**Table 1.** **Physical and baseline functional characteristics of bovine tracheal strips.**

| Control muscles Test muscles | | | |
| --- | --- | --- | --- |
| EFS studies^‡^ | | | |
| Weight, mg | Fresh | 13.9 ± 9.3 | 12.8 ± 3.9 |
|  | 24-h stored | 14.4 ± 9.0 | 11.4 ± 5.4 |
| Resting Force, g | Fresh | 0.5 ± 0.4 | 0.6 ± 0.1 |
|  | 24-h stored | 0.5 ± 0.4 | 0.7 ± 0.3 |
| Active force @64 Hz, g | Fresh | 21.8 ± 4.0 | 18.0 ± 0.2 |
|  | 24-h stored | 21.3 ± 8.6 | 17.7 ± 6.3 |
| Exogenous ACh studies^#^ | | | |
| Weight, mg * | Fresh | 10.6 ± 3.0 | 10.6 ± 0.7 |
|  | 24-h stored | 6.9 ± 2.3 | 7.0 ± 1.4 |
| Resting Force, g * | Fresh | 0.3 ± 0.1 | 0.6 ± 0.1 |
|  | 24-h stored | 0.7 ± 0.2 | 0.8 ± 0.2 |
| Active force @10^-4^M, g | Fresh | 23.7 ± 1.1 | 21.8 ± 2.0 |
|  | 24-h stored | 20.6 ± 1.8 | 21.0 ± 2.5 |
| ACh release studies^¶^ | | | |
| Weight, mg ** | Fresh | 27.5 ± 7.0 | 27.3 ± 7.3 |
|  | 24-h stored | 20.7 ± 5.4 | 20.3 ± 5.8 |
|  | 48-h stored | 18.7 ± 4.1 | 21.5 ± 4.9 |
| Resting Force, g | Fresh | 0.8 ± 0.4 | 0.9 ± 0.5 |
|  | 24-h stored | 0.7 ± 0.4 | 0.7 ± 0.4 |
|  | 48-h stored | 0.5 ± 0.3 | 0.7 ± 0.4 |
| Active force @4 Hz, g | Fresh | 20.9 ± 7.4 | 20.9 ± 7.8 |
|  | 24-h stored | 19.7 ± 6.2 | 18.2 ± 5.2 |
|  | 48-h stored | 15.7 ± 3.8 | 16.7 ± 3.3 |
| ACh release @4 Hz, disintegrations | Fresh | 1242 ± 542 | 1197 ± 563 |
|  | 24-h stored | 1586 ±1059 | 1658 ± 1456 |
|  | 48-h stored | 1352 ± 563 | 1604 ± 889 |

Data are mean ± SD; ‡, n=24 (fresh and stored); #, n=24 (fresh), 32 (stored); ¶, n= 32 (fresh), 26 (24-h stored), 16 (48-h stored). *, p<0.017 and **, p=0.004 stored vs. fresh; p>0.194 for all other comparisons fresh vs. stored; p>0.153 for all comparisons between control and test muscles; interaction between fresh/stored vs. control/test p>0.081 for all comparisons (ANOVA).

Figure 1:


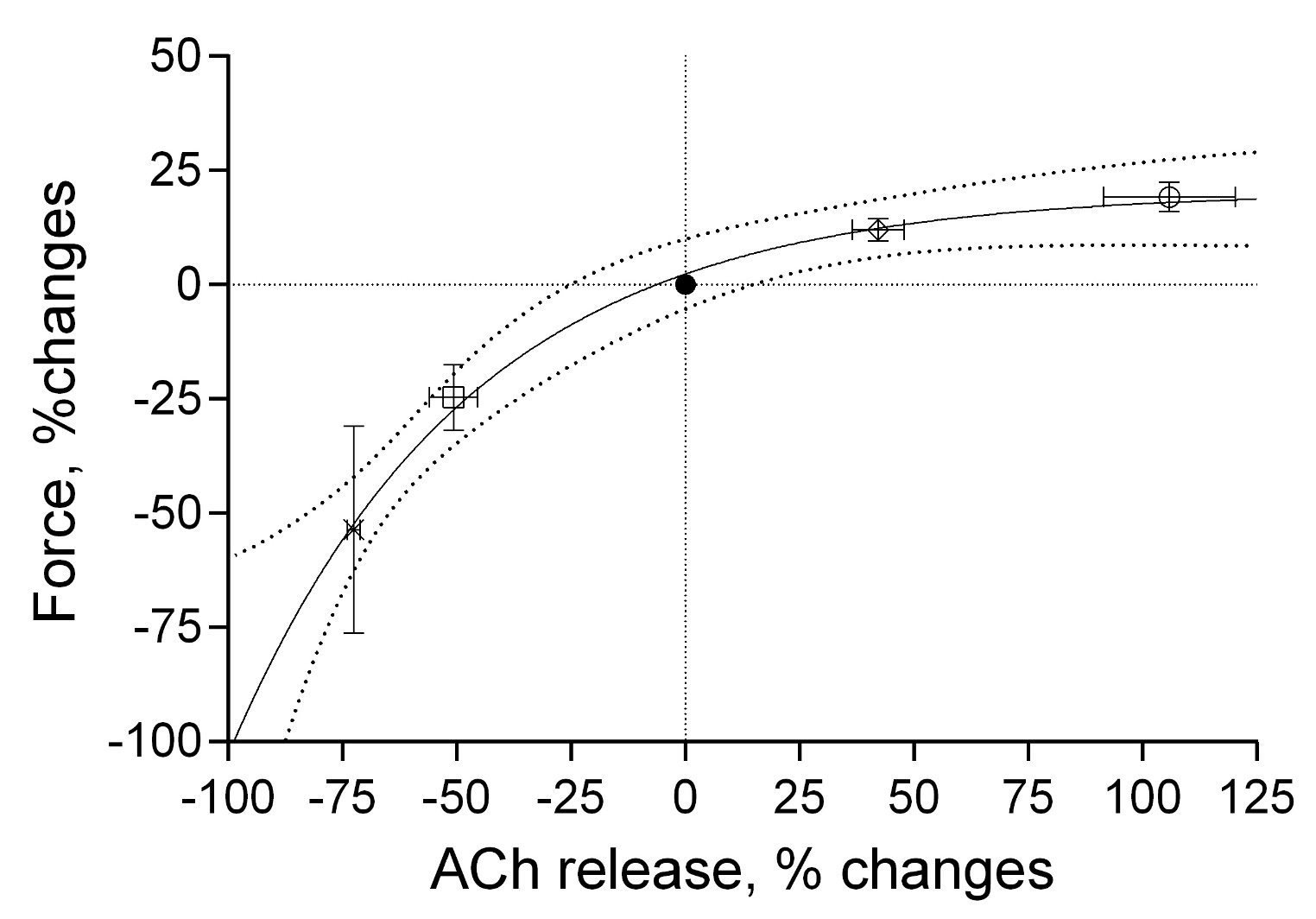


Figure 1. Relationships between ACh-release and isometric force at ES frequencies of 1 (x), 2 (□), 4 (●), 8 (◊), and 16 (○) Hz in 4 separate experiments. Data are mean and SD of percent changes from 4 Hz; continuous thick line is the regression model o$f the form y=\left( plateau-y_{0} \right)\bullet\left[ 1-e^{\left( -k\bullet x \right)} \right]$, interrupted lines are the 95% CI; r^2^=0.887.
